# Vasculitis masquerading as drug allergy: thinking outside the ‘adult’ box of possible diagnoses

**DOI:** 10.1186/1710-1492-8-S1-A15

**Published:** 2012-11-02

**Authors:** Marie-Elodie Sarre-Annweiler, Mariamma Joseph, Kyla J Hildebrand

**Affiliations:** 1 Division of Dermatology, Department of Medicine, Angers University Hospital, University of Angers, Angers, France; 2 Division of Clinical Immunology & Allergy, Department of Medicine, Schulich School of Medicine & Dentistry, Western University, London, Ontario, Canada; 3 Division of Cytology , Department of Pathology, Schulich School of Medicine & Dentistry, Western University, London, ON, Canada

## Case report

**Figure 1:**
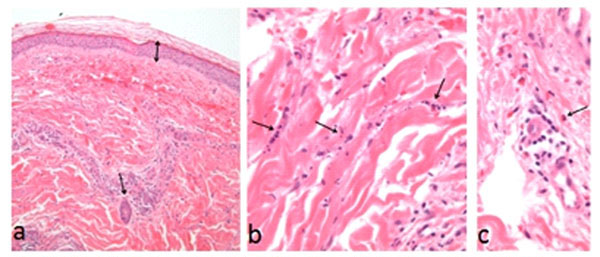
**Histological images of neutrophilic small-vessel leukocytoclastic vasculitis (skin punch biopsy from patient's leg). a.** Normal epidermis. On dermis, extravasated red cells and mild perivascular inflammation. **b,c.** Inflammatory cells (High power): neutrophils and nuclear dust (b), rare eosinophils (c).

A 32 year-old male presented with fever and pharyngitis. Amoxicillin was prescribed and 5 days into therapy he developed a petechial rash on the lower extremities, arthritis of the ankles, wrists and elbows, and loose stools. He completed the amoxicillin with no worsening of symptoms. A vasculitis assessment in the Internal Medicine Clinic found a slightly elevated ANA and normal ANCAs, hepatitis B/C/HIV serologies, CH50, C3, C4, rheumatoid factor, CBC, electrolytes, coagulation, urinalysis and chest X-ray. Skin biopsy confirmed a neutrophilic small-vessel leukocytoclastic vasculitis (Figure 1). The skin rash and arthritis resolved over the next 4-6 weeks with residual hyperpigmentation and scarring. The symptoms were attributed to a possible drug allergy to amoxicillin and avoidance was recommended.

**Figure 2:**
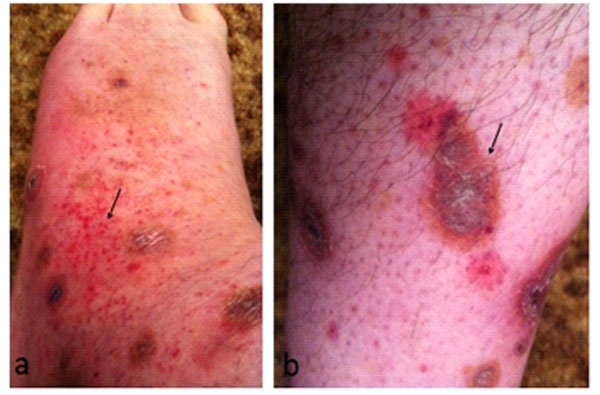
**Images of rash during the second episode of fever and pharyngitis.** a. Petechial rash on the lower extremeties. b. Residual hyperpigmentation and scarring.

Two months later, fever and pharyngitis recurred and a similar reaction occurred within 48 hours of azithromycin treatment (Figure 2). A referral was made the Adverse Drug Reaction clinic. IgE-mediated symptoms were absent. Previous treatments with penicillin were tolerated.

## Conclusions

Skin exanthems have a broad differential diagnosis. Henoch-Schonlein-Purpura (HSP) is a small vessel vasculitis with purpura, arthritis, and gastrointestinal symptoms with 90% of cases occurring in children. A dermatology referral was made and the current working diagnosis is HSP or polyarteritis nodosum (PAN) pending a repeat biopsy during the next acute flare. Skin exanthems are often attributed to concurrent medications. The clinical history in a drug allergy assessment is key in distinguishing hypersensitivity drug reactions from other causes including vasculitis. Drug allergy assessment can prevent unnecessary future antimicrobial avoidance in patients with skin exanthems.

